# The Transmodulation of HER2 and EGFR by Substance P in Breast Cancer Cells Requires c-Src and Metalloproteinase Activation

**DOI:** 10.1371/journal.pone.0129661

**Published:** 2015-06-26

**Authors:** Susana Garcia-Recio, Eva M. Pastor-Arroyo, Mercedes Marín-Aguilera, Vanessa Almendro, Pedro Gascón

**Affiliations:** 1 Department of Medical Oncology, Hospital Clínic, Barcelona, Spain; 2 Institut d’Investigacions Biomediques August Pi i Sunyer, Barcelona, Spain; 3 Department of Medicine, University of Barcelona, Barcelona, Spain; II Università di Napoli, ITALY

## Abstract

**Background:**

Substance P (SP) is a pleiotropic cytokine/neuropeptide that enhances breast cancer (BC) aggressiveness by transactivating tyrosine kinase receptors like EGFR and HER2. We previously showed that SP and its cognate receptor NK-1 (SP/NK1-R) signaling modulates the basal phosphorylation of HER2 and EGFR in BC, increasing aggressiveness and drug resistance. In order to elucidate the mechanisms responsible for NK-1R-mediated HER2 and EGFR transactivation, we investigated the involvement of c-Src (a ligand-independent mediator) and of metalloproteinases (ligand-dependent mediators) in HER2/EGFR activation.

**Results and Discussion:**

Overexpression of NK-1R in MDA-MB-231 and its chemical inhibition in SK-BR-3, BT-474 and MDA-MB-468 BC cells significantly modulated c-Src activation, suggesting that this protein is a mediator of NK-1R signaling. In addition, the c-Src inhibitor 4-(4’-phenoxyanilino)-6,7-dimethoxyquinazoline prevented SP-induced activation of HER2. On the other hand, SP-dependent phosphorylation of HER2 and EGFR decreased substantially in the presence of the MMP inhibitor 1–10, phenanthroline monohydrate, and the dual inhibition of both c-Src and MMP almost abolished the activation of HER2 and EGFR. Moreover, the use of these inhibitors demonstrated that this Src and MMP-dependent signaling is important to the cell viability and migration capacity of HER2+ and EGFR+ cell lines.

**Conclusion:**

Our results indicate that the transactivation of HER2 and EGFR by the pro-inflammatory cytokine/neuropeptide SP in BC cells is a c-Src and MMP-dependent process.

## Introduction

The cellular and non-cellular components of the tumor microenvironment shape tumor evolution[1]. Among the components of the tumor microenvironment, the nervous system and the neuropeptides secreted by non-neuronal (i.e., by modulating immune cells) and neuronal cells appear to have a direct and indirect effects on tumor progression [2]. This is the case of neurokinin 1 receptor (NK-1R) (*TACR1* gene) and its preferential ligand substance P (SP) (*TAC1* gene), a pro-inflammatory cytokine and neuropeptide that belongs to the family of tachykinins [3, 4]. This family consists of SP, neurokinin A (NKA) and neurokinin B (NKB), encoded by the *TAC1* (SP and NKA) or *TAC3* (NKB) genes [5], and the recently discovered hemokinins and endokinins encoded by the *TAC4* gene [5–7]. Specifically, NK-1R is a G-protein coupled receptor (GPCR) which, together with SP, is expressed in the central nervous, gastrointestinal, and immune systems, and is involved in cellular responses such as pain transmission, paracrine and endocrine secretion, vasodilation, angiogenesis and modulation of cell proliferation [5, 8–11]. SP not only signals through NK-1R; it can also bind (with lower affinity) to additional tachykinin receptors like neurokinin 2 receptor (NK-2R) and neurokinin 3 receptor (NK-3R) encoded by the *TACR2* and the *TACR3* gene respectively [5, 12].

Despite their physiological functions, G proteins can also activate pathways related to cellular proliferation and survival in several types of cancer cell through secondary messengers and receptors, as in the case of NK-1R [13–15]. This receptor is expressed on the cell surface of many cancer cell types like breast [16–19], pancreatic [20], colon [21, 22], and laryngeal cancer cells [23], glioblastoma [22], acute lymphoblastic leukemia [5, 24], and melanoma [5]. NK-1R signaling can activate tyrosine kinase receptors (RTKs) like EGFR and HER2 [25–27]. The RTK family shares a similar structure, and the receptors belonging to the ErbB family (EGFR, HER2, HER3, and HER4) are driver oncogenes in different types of cancer [28, 29]. Several reports have shown the involvement of the non-receptor protein tyrosine kinase c-Src and metalloproteinases (MMPs) in the GPCR-mediated activation of ErbB receptors [30–32]. Activated c-Src can bind to the cytoplasmic tail of EGFR and HER2 and phosphorylate tyrosine residues; therefore, c-Src activation may lead to the triggering of ErbB receptors in a ligand-independent manner [30, 31]. The signal transduction by G-proteins may also enhance ligand-mediated EGFR activation by stimulating MMPs synthesis and secretion and favoring the shedding of membrane-anchored ligands [14, 33].

The interaction of GPCRs and RTKs has a prominent role in various physiological processes [13, 34, 35], but it is also involved in pathologic conditions since its deregulation can drive tumorigenic processes [14]. We previously identified SP as a key modulator of the steady state of HER2 and EGFR, with the functional consequence of enhanced tumor aggressiveness and tumor progression, and alterations in the cellular responses to apoptotic stimuli [27]. In the present study, we aimed to identify the mechanisms involved in the transactivation of HER2 and EGFR by SP in BC cells. Focusing on the involvement of ligand-independent and dependent mediators, we conclude that the transmodulation of HER2 and EGFR in response to SP is a c-Src and MMP-dependent mechanism.

## Materials and Methods

### Cell lines and reagents used in the study

The following cell lines were purchased from American Type Culture Collection and were cultured in accordance with the instructions: MDA-MB-453, BT-474, SK-BR-3, MDA-MB-231, and MDA-MB-468. The cultures were incubated at 37°C in a humidified 5% CO_2_ atmosphere and the cells were serum starved overnight before experiments, unless otherwise specified. For some proliferation experiments, cells were grown in a complete growth medium plus fetal bovine serum (FBS), as specified in the methods section. The authenticity of all the cell lines used in this study was validated by single locus short tandem repeats (STR) typing (Bio-Synthesis, Inc.).

Insulin (Cat# I-9278), Substance P (Arg-Pro-Lys-Pro-Gln-Gln-Phe-Phe-Gly-Leu-Met-NH2) (Cat# S1136), and MMP inhibitor 1–10, phenanthroline monohydrate (Cat# P9375) were obtained from Sigma-Aldrich. NK-1R antagonist L-733,060 was obtained from Tocris (Cat# 1145) and c-Src inhibitor 4-(4′-phenoxyanilino)-6,7-dimethoxyquinazoline from Calbiochem (Cat# 567805). All were prepared in accordance with the instructions.

### Time-course studies

To determine the effects of SP treatment on c-Src activation, cells were seeded in 100 mm culture dishes, grown until 80% confluence, serum starved for 24 hours, and then treated at the indicated times with 100 nM of SP. After each treatment, the cells were washed twice in cold PBS and rapidly frozen until protein extraction. To determine the effects of SP in the presence of c-Src, inhibitor cells grown until 80% confluence were serum starved for 4 hours, and treated for 20 hours with c-Src inhibitor 4-(4′-phenoxyanilino)-6,7-dimethoxyquinazoline (1μM). To determine the effects of SP in the presence of the MMP inhibitor 1–10, phenanthroline monohydrate, cells were serum starved for 16 hours and treated with phenanthroline monohydrate (7μM) for 4 hours. Finally, to determine the effects of SP in the presence of c-Src and MMP inhibitors, cells were serum starved for 4 hours and treated with c-Src inhibitor 4-(4′-Phenoxyanilino)-6,7-dimethoxyquinazoline for 16 hours. At this point phenanthroline monohydrate was added to the cells, and both inhibitors were incubated for 4 additional hours. The control group without treatment was serum starved for 24 hours. Subsequently, the cells were treated with SP 100 nM for 6, 10 and 15 min. After the treatment, the cells were washed twice in cold PBS, and rapidly frozen until protein extraction. The experiments with each cell line were repeated at least twice to ensure the reproducibility of the data. In all cases, the corresponding dose of DMSO or MetOH (never above 0.1% v/v) was added to the control points.

### Overexpression of *TACR1*

The *TACR1* expression vector pcDNA3.1(+)-*TACR1* was obtained from the University of Missouri-Rolla cDNA Resource Center. The empty vector pcDNA3.1(+) was generated by removing *TACR1* insert. The constructs were transfected into the MDA-MB-231 by AMAXA nucleofection (Amaxa, Germany). Briefly, around 2x10^6^ of MDA-MB-231 cells were resuspended in 100 μl of Nucleofector V solution (Amaxa, Germany) and 5 μg of pcDNA3.1(+) or pcDNA3.1(+)-*TACR1* vectors were added to the cell suspension. The electrogene transfer was conducted using the Amaxa Nucleofector system program X-13. The selection of positive clones was performed by antibiotic selection with G418 (Invitrogen, CA) (1200 μg/ml) for at least 2 weeks, and additional enrichment by Fluorescent Activated Cell Sorting (FACS).

### Inhibition of NK-1R with L-733,060 antagonist

To inhibit NK-1R signaling, cells cultured until 70% confluence were serum starved for 5 hours and then treated with 20 μM (SK-BR-3 and BT-474) and 30 μM (MDA-MB-453) of NK-1R antagonist L-733,060 during 24h. For the simultaneous inhibition of the three receptors NK-1R, NK-2R and NK-3R cells were also treated with MEN 10376 (30 μM, NK-2R antagonist) and SB 218795 (20 μM, NK-3R antagonist). After the treatment, the cells were washed twice in cold PBS, and rapidly frozen until protein extraction. The experiments with each cell line were repeated at least three times to ensure the reproducibility of the data, and all quantitative measurements were generated from three or more replicates. The statistical significance of the data was analyzed by *t*-test (two-tailed). *P* values < 0.05 were considered statistically significant.

### Western blot

For protein extraction, cells were lysed in ice-cold radioimmunoprecipitation assay buffer (RIPA) (Tris-HCl 50 mM, pH 7.4; NP-40 1%; Na-deoxycholate, 0.25%; NaCl 150 mM; EDTA 1 mM; PMSF 1 mM; proteinase inhibitors; Na3VO4 1 mM and NaF 1 mM) and sonicated for 10 seconds. After centrifugation (13000 rpm from 5 min) supernatants were quantified for protein content. Equal amounts of proteins were separated by SDS-PAGE and electrophoretically transferred to polyvinylidene difluoride membranes (BioRad Laboratories, CA), blocked with 5% milk in PBS for 1 hour, incubated overnight with the corresponding primary antibodies: phospho-EGFR Tyr1068 (Cell Signaling, MA, Cat# 2234) at 1:1000 dilution, phospho-HER2 Tyr1248 (Abcam, UK, Cat# ab5654) at 1:500 dilution, phospho p42/44-MAPK (Cell Signaling, MA, Cat# 9101S) at 1:1000 dilution, phospho-Src Family (Tyr416) at 1:500 dilution (Cell Signaling, MA, Cat# 2101), Src (Cell Signaling, MA, Cat# 2109S) at 1:1000 dilution and then, one hour with goat anti mouse HRP-conjugated (Amersham, NJ, Cat# NXA931) or goat anti rabbit HRP-conjugated (GE Healthcare Amersham, NJ, Cat# NA934V) at 1:2000. To confirm equal protein loading, membranes were incubated with α-tubulin (Cell Signaling, MA, Cat# 2144) or α-actin (Sigma Aldrich, MO, Cat#A2066) antibodies at 1:2000 dilution as internal control. Chemiluminiscence on membranes was detected after ECL treatment (GE Healthcare Amersham, NJ, Cat# RPN2209) and image capture was performed with a Fujifilm LAS3000 imaging system. The Image Gauge software was used for the densitometric quantification of each protein. Correct Mr was compared with pre-stained protein standards (BioRad Laboratories, CA, Cat# 161–0374). The experiments with each cell line were repeated at least three times to ensure the reproducibility of the data and all quantitative data were generated from three or more replicates. The statistical significance of the data was analyzed by *t*-test (two-tailed). *P* values < 0.05 were considered statistically significant.

### Cell viability assay

Cell viability was assessed in subconfluent cell cultures that were incubated for 24 hours with IC50 of MMP inhibitor 1–10, phenanthroline monohydrate (16μM in SK-BR-3 and 8μM in MDA-MB-468), c-Src inhibitor 4-(4′-phenoxyanilino)-6,7-dimethoxyquinazoline (6μM in SK-BR-3 and 50 μM in MDA-MB-468), or L-733,060 antagonist (9μM in SK-BR-3 and 10μM in MDA-MB-468) or with the IC50 combinations drug: MMP inhibitor + Src inhibitor (14μM+5 μM in SK-BR-3; 6μM+40 μM in MDA-MB-468), MMP inhibitor + L-733,060 (14μM+9 μM in SK-BR-3; 6μM+8 μM in MDA-MB-468), Src inhibitor + L-733,060 (5μM+9 μM in SK-BR-3; 40μM+8 μM in MDA-MB-468) and MMP inhibitor + Src inhibitor + L-733,060 (14 μM+5μM+ 9 μM in SK-BR-3; 6μM+40μM+ 8 μM in MDA-MB-468) in serum free medium. Briefly, cells were seeded in 96-well plates at a density of 1 ×10^5^ cells/well and allowed to attach overnight. After treatment, cells were washed and the cell viability was determined with Calcein Assay Kit (Molecular Probes). For viable fluorescent cells detection, 100 μl of calcein working solution were added to each well and an additional 100 μl of PBS, yielding 200 μl per well containing 2μM of calcein. The cells were incubated for 45 minutes at 37°C in a humidified 5% CO_2_ atmosphere and then, the plate was read on a Synergy HT Multi-Detection Microplate Reader (BioTek) at 485±10 nm (excitation optical filter) and 530±12,5 nm (emission optical filter). Different doses were assessed in sixtiplicate. In all cases, the corresponding dose of DMSO or MetOH (never above 0.1% v/v) was added to the control points. Assay values for controls were taken as 100% of viability, and the viability at each treatment point were calculated relative to controls by the formula: %Live Cells = (F(530)_sam-_F(530)_min)_/F(530)_max_-F(530)_min_) x 100% according to the manufacturer’s instructions.

### Cell migration assay

Cell migration was assessed in culture cells not greater than 80% confluence and serum starved 24h prior to assay. A total of 1 × 10^6^ harvest cells in 50 μl of serum-free medium /well were plated in the top chamber of the transwell with a noncoated polyethylene terephthalate (PET) membrane, 8.0 μm pore size (Cultrex, 96 well cell migration Assay,Trevigen) and then, 50 μl serum-free medium with MMP inhibitor (16μM in SK-BR-3 and 8μM in MDA-MB-468), c-Src inhibitor (6μM in SK-BR-3 and 50 μM in MDA-MB-468), or L-733,060 antagonist (9μM in SK-BR-3 and 10μM in MDA-MB-468) or with the combination of MMP inhibitor + Src inhibitor (14μM+5 μM in SK-BR-3; 6μM+40 μM in MDA-MB-468) or without inhibitors were added. Complete growth media with 10% FBS with or without inhibitors was added to the bottom chamber as a chemoattractant. After incubation for 24hours, the top and the bottom chamber were washed with 200 μl of wash buffer and were added 100 μl of cell dissociation Solution/Calcein AM (2μM) to each well of bottom chamber, and incubated at 37°C in CO_2_ incubator for 1h. The bottom assay chamber was read at 485nm exciation, 520 nM emission on a Synergy HT Multi-Detection Microplate Reader (BioTek). Different doses were assessed in sixtiplicate. In all cases, the corresponding dose of DMSO or MetOH (never above 0.1% v/v) was added to the control points. Assay values for controls without inhibitors were taken as 100% of migration, and the viability at each treatment point were calculated relative to their controls.

### Statistical analysis

Statistical analysis of the results was performed by ANOVA with Tukey's Multiple Comparison post-hoc test and *t*-test (two-tailed). Statistical significance was considered since *P* values less than 0.05.

## Results

### The neuropeptide/proinflammatory mediator SP activates c-Src in BC cell lines

**Fig 1:**
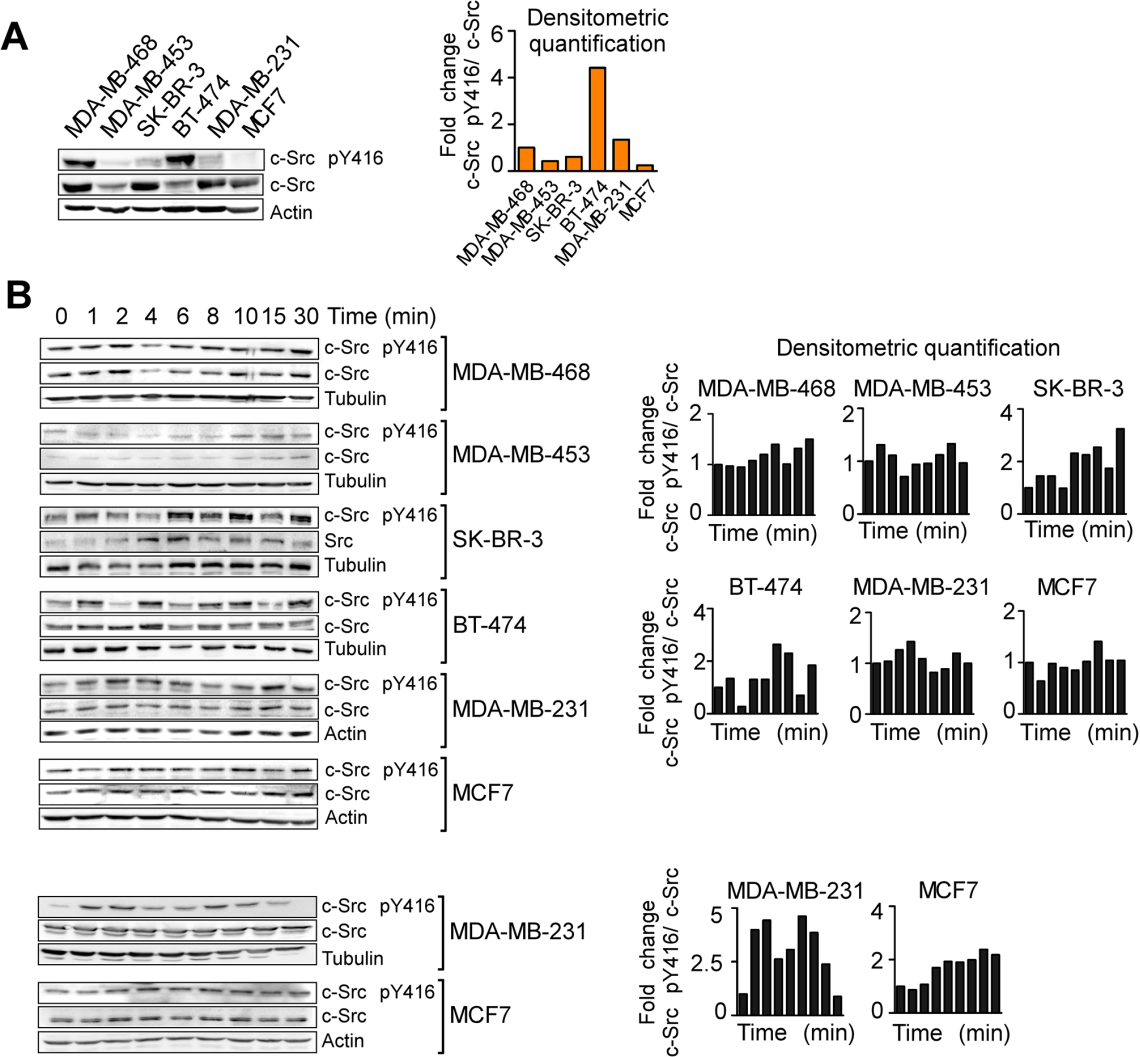
c-Src phosphorylation levels at baseline or under SP treatment. Representative images of Western blots corresponding to experiments showing (A) the basal levels of c-Src Y416 phosphorylation in BC cell lines and (B) c-Src (Y416) phosphorylation at 0, 1, 2, 4, 6, 8, 10, 15 and 30 minutes after SP 100 nM stimulation in different BC cell lines. The blot was standardized to c-Src levels. The plots accompanying each panel show the densitometric quantification of Western blots (the ratio of intensities of the bands corresponding to phospho-Y416 and total c-Src) relative to the expression of tubulin or actin, which was used to ensure equal protein loading.

We first investigated whether SP used the c-Src protein as a cell-signaling mediator in BC cells, as previously shown in other cell types [36, 37]. First, we checked a panel of BC cell lines under basal conditions without stimulation (Fig 1A) and we detected different levels of phosphorylated c-Src protein relative to total levels. Second, using time-course studies, we observed that SP treatment induced the phosphorylation of c-Src Tyr416 (indicative of Src activation [38, 39]) at the indicated time points (Fig 1B) in all the cell lines, including the HER2 negative cell lines MDA-MB-231 or MCF7. Some lines have more pronounced phosphorilation than others, and this activation is not consistent in all time points used because NK-1R activation by SP is a cyclic activation as we previously described [16]. For these reason, the activation of c-Src Tyr416 within this time frame was consistently observed in all the replicates conducted, although the exact time point and intensity of maximum activation varied. Lower activation of c-Src was found after SP treatment in the MDA-MB-453 cell line and was slightly pronounced in MDA-MB-468 line, probably due to the very low or very high basal levels of c-Src (phosphorylated and total protein) [30], respectively.

### The overexpression or inhibition of NK-1R modulates c-Src activity in BC cell lines

**Fig 2:**
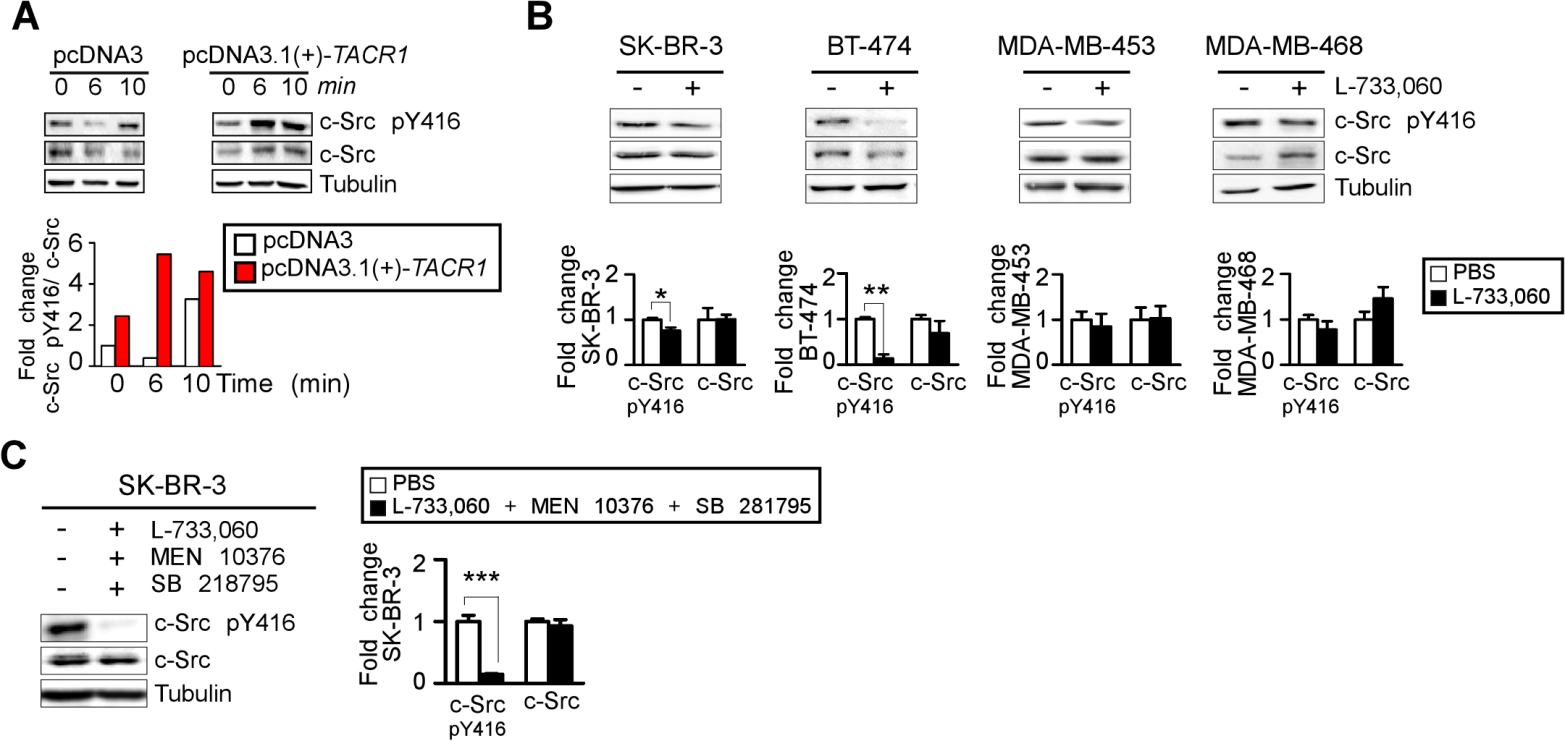
NK-1R contributes to c-Src activation in BC cell lines. (A) The contribution of NK-1R to the activation of c-Src Y416 phosphorilation protein in the MDA-MB-231 cell line transfected with pcDNA3.1(+)-*TACR1* or empty vector and treated for 6 and 10 minutes with SP 100 nM; (B) the effects of single NK-1R inhibition during 24h with L-733,060 (20 μM (SKBR3 and BT-474), 30 μM (MDA-MB-453)) or (C) combined NK-1R, NK-2R and NK-R3 inhibition during 24h with L-733,060 (20 μM), MEN 10376 (30 μM) and SB218795 (20 μM), respectively on c-Src (Y416). The blot was standardized to c-Src levels. All quantitative data are generated from a minimum of 3 replicates and are presented as mean + S.D. and compared by *t*-test (two-tailed) as * *P*<0.05, ** *P*<0.01 and *** *P*<0.001.

We previously reported that the stable transfection of NK-1R into the HER2-negative MDA-MB-231 cell line can be used as a tool to study the mechanism by which SP contributes to the persistent transmodulation of the ERBB receptors [17]. These previous results demonstrated that the overexpression of NK-1R enhanced SP-mediated HER2 activation even in a HER2- negative and NK-1R-low cell line, the main reason we selected that particular cell line [17]. To further confirm the involvement of NK-1R in c-Src activation, in the present study we investigated the effects of NK-1R overexpression on c-Src activation in the MDA-MB-231 cell line. The MDA-MB-231 cells were transfected with pcDNA3.1(+)-*TACR1* or the empty vector pcDNA3.1(+) and treated with SP 100 nM at 6 and 10 minutes. We observed that the basal levels of p-Src Y416 (at point 0, red bar) were 2.5-fold higher in the MDA-MB-231 cells overexpressing NK-1R compared to the control cells (at point 0, open bar) (Fig 2A). Then, in pcDNA3.1(+) transfected cells (left) (representing a basal situation), the treatment with SP for 10 minutes further increased the phosphorylation of c-Src Tyr416 (3.26 fold increased, open bar) as we found in MDA-MB-231 cell line in Fig 1. On the other hand, in the MDA-MB-231 cells overexpressing NK-1R (pcDNA3.1(+)-*TACR1*), the treatment with SP for 6 or 10 minutes further increased the phosphorylation of c-Src Tyr416 (5.5- and 4.6-fold, respectively, red bar) and in all cases were expressed by ratio of phospho/total protein (Fig 2A).

We next analyzed the effects of NK-1R inhibition on c-Src activation. The HER2+ SK-BR-3, BT-474, MDA-MB-453, and the EGFR+ MDA-MB-468 cell lines were treated with the NK-1R antagonist L-733,060 for 48 hours. NK-1R antagonism significantly reduced c-Src phosphorylation at Tyr416 in SK-BR-3 and BT-474 cell lines while a non-significant trend towards inhibition was observed in the MDA-MB-453 and MDA-MB-468 cell line with the lowest or highest levels of c-Src (phosphorilated and total protein), respectively (Fig 2B), so, it is not surprising to observe fewer changes in cell lines which steady state of c-Src (both, the phosphorylated and total protein) is already low or high. Since SP can also bind with lower affinity to NK-2R and NK-3R receptors, we next investigated the effects of the triple chemical inhibition of NK-1R, NK-2R, and NK-3R with L-733,060, MEN 10376, and SB 218795 inhibitors respectively. We observed that the triple inhibition of SP receptors cause a dramatic downregulation of c-Src phosphorylation (Fig 2C), indicating that c-Src is indeed triggered by tachykinin signaling in BC cells.

### The transactivation of HER2 and EGFR by SP is dependent on c-Src and MMPs

**Fig 3:**
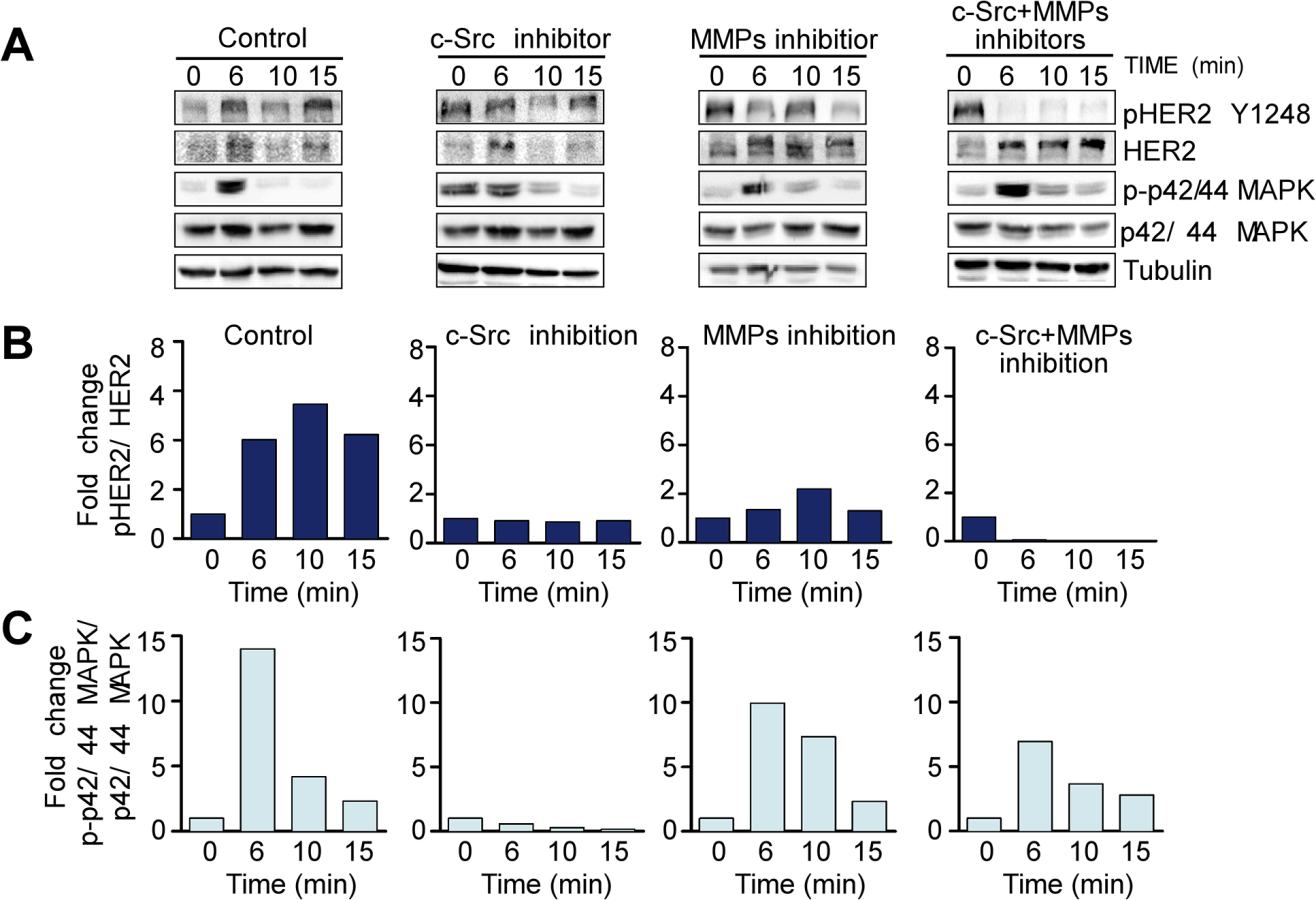
SP transmodulates HER2 by c-Src and MMP-dependent mechanisms in SKBR3 cell line. (A) Representative images of Western blots evaluating the effects of the single or combined inhibition of c-Src (Y416) with 4-(4′-phenoxyanilino)-6,7-dimethoxyquinazoline (1μM) and MMPs with 1–10, phenanthroline monohydrate (7μM) on the activation of HER2 and p42/44 MAPK triggered by SP 100 nM for 6, 10 and 15 minutes. The plots show the densitometric quantification of the Western blots on phosphorylated (denoted by p-) HER2 (B) and p42/44 MAPK (C) relative to the expression of tubulin, which was used to ensure equal protein loading. Western blots are representative of at least two independent experiments.

To investigate the role of c-Src in SP-mediated HER2 and EGFR activation [30] we next performed time-course studies with SP in the presence of the c-Src inhibitor 4-(4′-phenoxyanilino)-6,7-dimethoxyquinazoline [40]. Inhibition of c-Src activity in the HER2 positive SK-BR-3 cell line significantly blocked SP-induced phosphorylation of HER2 Tyr1248 compared to control cells (Fig 3A and 3B). HER2 transactivation by SP was also substantially inhibited in the presence of the MMP inhibitor 1–10, phenanthroline monohydrate, and almost completely abolished after the inhibition of both pathways, suggesting that the transactivation of HER2 by SP in BC cells is a c-Src and MMP-dependent process (Fig 3A and 3B). SP signaling activates the mitogen-activated protein kinase (MAPK) pathway [10, 26, 41]; therefore, the phosphorylation of p42/44 MAPK was used to control of NK-1R downstream activation. For this reason, the phosphorylation of p42/44 MAPK was not always reduced in the presence of c-Src and MMP inhibitors (Fig 3A and 3C), since the activation of the MAPK pathway can be triggered by both NK-1R and ERBB signaling [14, 37].

**Fig 4:**
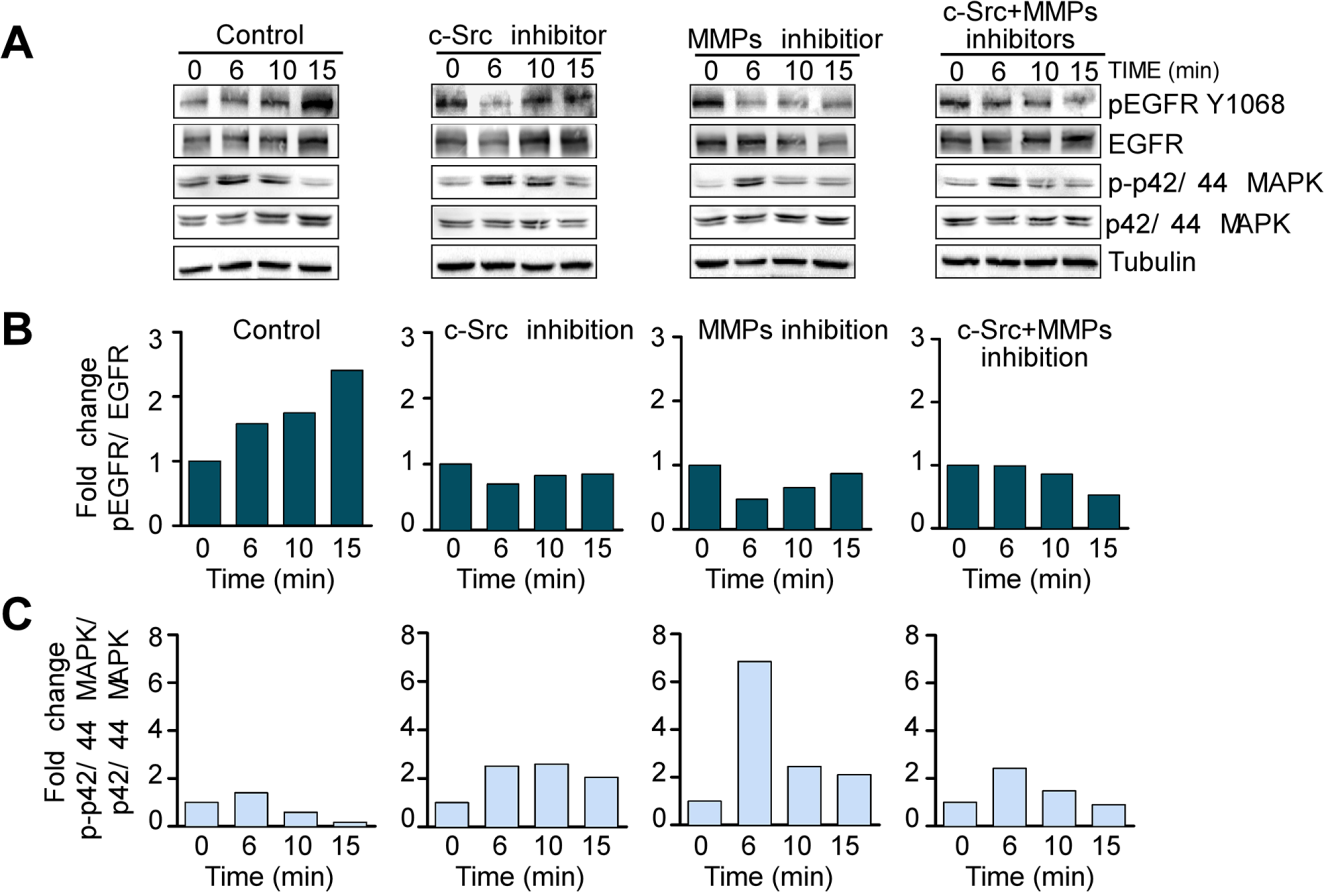
SP transmodulates EGFR by c-Src and MMP-dependent mechanisms in the MDA-MB-468 cell line. (A) Representative images of Western blots evaluating the effects of the single or combined inhibition of c-Src (Y416) with 4-(4′-phenoxyanilino)-6,7-dimethoxyquinazoline (1μM) and MMPs with 1–10, phenanthroline monohydrate (7μM) on the activation of EGFR and p42/44 MAPK triggered by SP 100 nM for 6, 10 and 15 minutes. The plots show the densitometric quantification of the Western blots on phosphorylated (denoted by p-) EGFR (B) and p42/44 MAPK (C) relative to the expression of tubulin, which was used to ensure equal protein loading. Western blots are representative of at least two independent experiments.

To determine whether the transmodulation of EGFR by SP was also dependent on c-Src and MMPs in BC cells, we performed similar experiments in the EGFR positive cell line MDA-MB-468. In the control situation, addition of SP increased EGFR phosphorylation at 6 min (1.17-fold), 10 min (1.45-fold) and particularly at 15 min (2.61-fold). No increase occurred in the presence of the inhibitors (alone or in combination) under SP treatment, as we observed in the western blot and densitrometic quantification of phospho EGFR/EGFR ratio (Fig 4A and 4B). In particular, c-Src inhibition significantly decreased the capability of SP to induce EGFR phosphorylation. Similarly, MMP inhibition affected the phosphorylation of EGFR induced by SP, as did the concomitant inhibition of both c-Src and MMPs, especially with both c-Src and MMPs inhibitor treatment at 15 min point (0.46 fold decrease) compared with point 0 (Fig 4B, right diagram).We also observed that c-Src inhibition significantly decreased the capability of SP to induce EGFR phosphorylation. Similarly, MMP inhibition affected the phosphorylation of EGFR induced by SP, as did the concomitant inhibition of both c-Src and MMPs, especially with both c-Src and MMPs inhibitor treatment at 15 min point (0.46 fold decrease) compared with the point 0, right diagram (Fig 4B). As before, the inhibition of c-Src and MMPs in this cell line did not block MAPK signaling due to its modulation by both NK-1R and RTKs (Fig 4A and 4C).

Taken together, these data demonstrate that SP-mediated HER2 and EGFR activation is a c-Src and MMP-dependent process in BC cells.

### The inhibition of c-Src, MMPs and NK-1R decreases cell viability and migration of breast cancer cells

**Fig 5:**
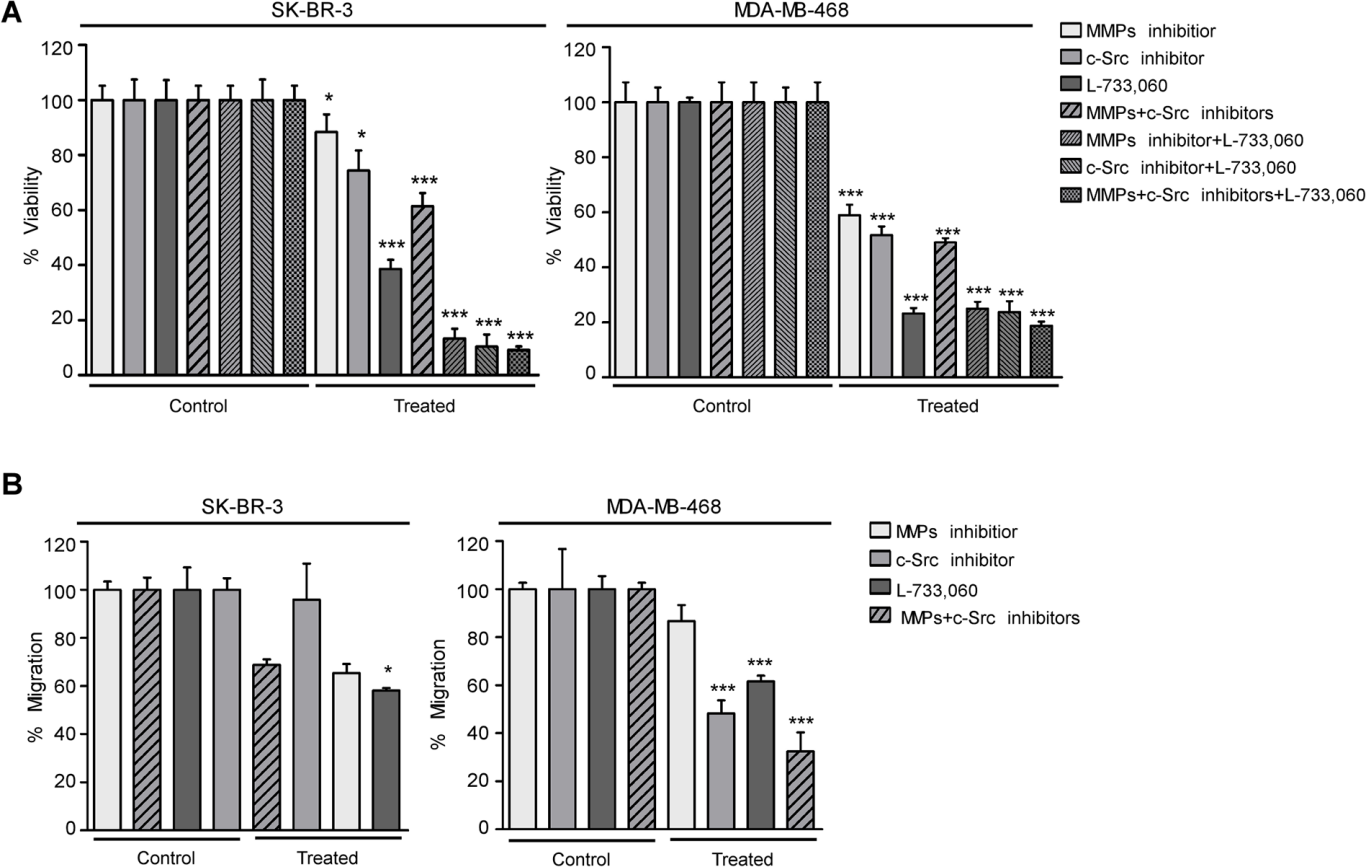
Blockade of c-Src, MMPs or NK-1R inhibits tumor cell viability and migration in SK-BR-3 and MDA-MB-468 cells. (A) Cell viability quantification of SK-BR-3 cells and MDA-MB-468 cells treated for 24h with IC50 values of MMP inhibitor, c-Src inhibitor, L-733,060 antagonist or with the combinations of drugs: MMP inhibitor + Src inhibitor, MMP inhibitor + L-733,060), Src inhibitor + L-733,060 and MMP inhibitor + Src inhibitor + L-733,060 in serum free medium. After 24h, the cells were treated with calcein (2 μM) for 45 min and calcein AM fluorescence was measured to determine cell viability. (B) Migration rate quantification of SK-BR-3 cells and MDA-MB-468 cells treated for 24h with IC50 values of MMP inhibitor, c-Src inhibitor, L-733,060 antagonist or with the combination of MMP inhibitor + Src inhibitor in serum-free medium. After 24h, detection of cell migration was quantified using calcein AM. Results are represented as mean of % viability or % migration ± SD. All the quantitative data are for a minimum of 6 replicates. Significant differences by ANOVA with Tukey Multiple Comparison post-hoc test are indicated as * P<0.05, ** P<0.01 and *** P<0.001.

To study the role of c-Src, MMPs, and NK-1R in cell viability and migration capacities, we treated the HER2+ SK-BR-3 and EGFR+ MDA-MB-468 cell lines with NK-1R antagonist L-733,060, c-Src inhibitor 4-(4′-phenoxyanilino)-6,7-dimethoxyquinazoline and MMP inhibitor 1–10, phenanthroline monohydrate. Cell viability was significantly decreased, above all under NK-1R antagonist in both cell lines (Fig 5A). Inhibition of c-Src and MMPs activity also significantly decreased cell viability and was almost completely abolished after the combination of each drug with NK-1R antagonist and after triple inhibition.

Of particular note, the migration rate of MDA-MB-468 significantly decreased under c-Src inhibitor, L-733-060 antagonist and after the combination of c-Src and MMPs inhibitor (Fig 5B); however, only L-733-060 significantly decreased the migration rate of SK-BR-3 cells. This finding suggests that the cells’ migration capacity was partially mediated through c-Src and NK-1R signaling in MDA-MB-468 and mainly by NK-1R signaling in SK-BR-3 cells (Fig 5B).

## Discussion

Tachykinins are pro-inflammatory mediators/neuropeptides that contribute to tumor progression by modulating the properties of both cancer and stromal cells. In previous work, we showed that SP contributes to BC progression by modulating the activity of oncogenic receptors like HER2 and EGFR, thus influencing tumor responses to targeted therapies designed to inhibit these receptors [16]. In the present study, we show that SP triggers HER2 and EGFR activation by activating c-Src and MMPs.

**Fig 6:**
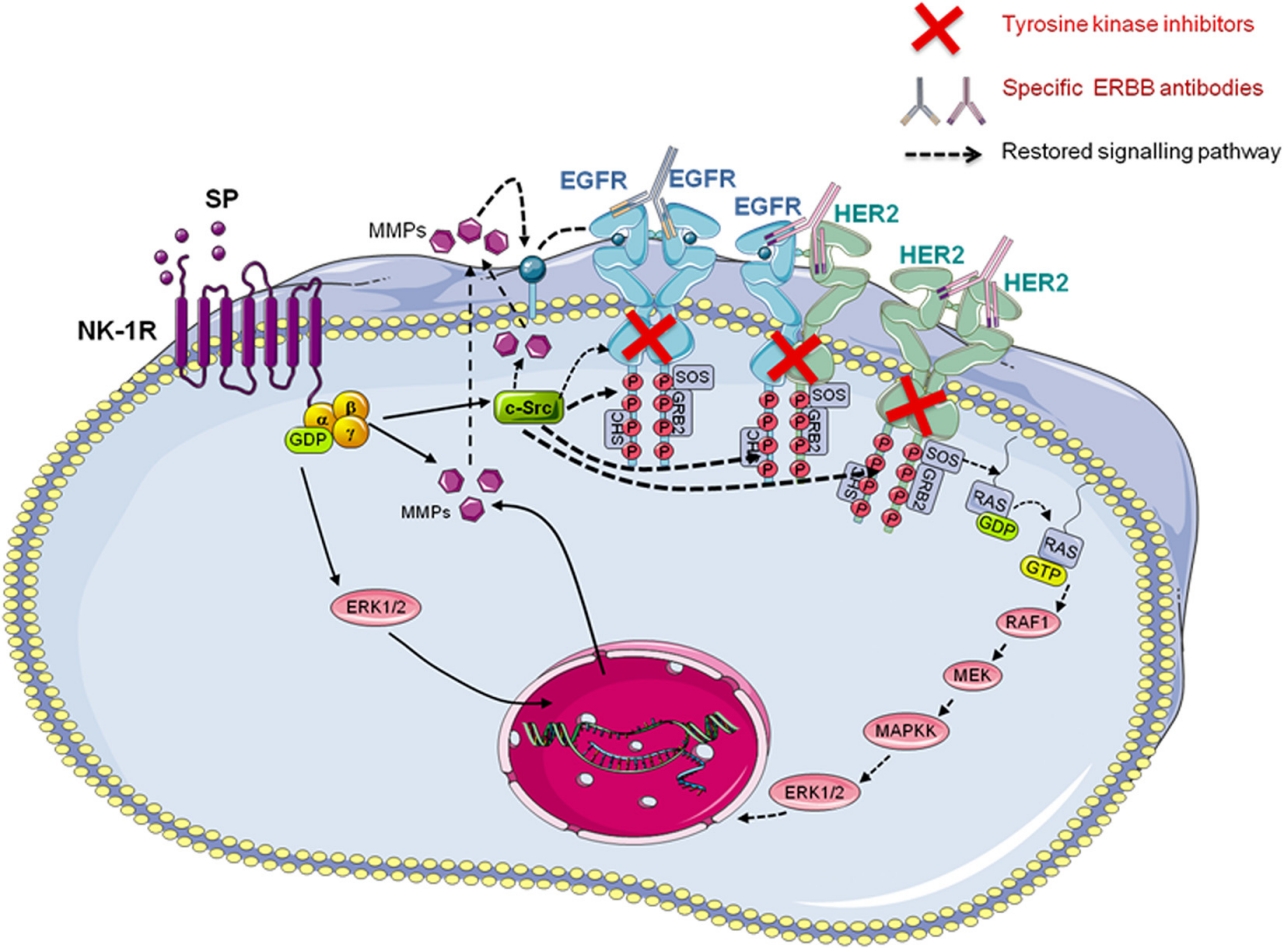
Proposed model of EGFR and HER2 transactivation by SP. NK-1R induces signal transduction through the activation of G proteins. Heterotrimeric G proteins consist of three different subunits: the Gα subunit that binds GDP/GTP, and the Gβ and Gγ subunits that form the Gβγ complex. The binding of an agonist (in this case Substance P) to NK-1R induces the activation of G proteins which in turn induce their own signaling cascade, such as the activation of the MAPK pathway or the phosphorylation of c-Src. The activation of the MAPK pathway in turn contributes to raising MMP secretion, which increases the cleavage of membrane-anchored ligands that in turn will bind the EGFR receptor. On the other hand, c-Src directly phosphorylates the cytoplasmic tails of both EGFR and HER2, allowing the binding of scaffold proteins that will further activate signal transduction. Based on our results, we propose that this direct phosphorylation of the cytoplasmic tails of EGFR and HER2 by c-Src may overcome the effects of tyrosine kinase inhibitors or antibodies or molecules against extracellular domains [17], since c-Src may act independently of EGFR and HER2 tyrosine kinase activity. *This figure has been made using Servier Medical Art* collection (http://creativecommons.org/licenses/by/3.0).

The modulation of the steady state of RTKs like HER2 and EGFR by neuropeptides such as SP can influence the clinical response of a tumor [17]. Although the oncogenic addiction to RTKs is therapeutically exploited for BC treatment, the transmodulation of RTKs by SP and other neuropeptides and pro-inflammatory mediators [42, 43] can influence the cancer cell response to RTK inhibitors since it serves as a mechanism for RTK activation in a ligand-independent way [14]. The protein tyrosine kinase c-Src can directly phosphorylate Tyr residues in the kinase domain HER2 [30, 32] and the cytoplasmic tail of EGFR [31], allowing the formation of stable homo- or heterocomplexes with other receptors or the binding of scaffold proteins and the activation of signal transduction. In addition, activated RTKs will reciprocally activate c-Src, thereby creating a positive regulatory loop. This overactivation may contribute to the permanent signaling through the RTKs and the maintenance of multiple signaling pathways downstream of the receptor [44]. Then, the transactivation of these receptors by c-Src-dependent mechanisms may contribute to the persistence of RTK-related signaling pathways even in the presence of tyrosine kinase inhibitors or antibodies against extracellular domains of these receptors (Fig 6).

It is known that the c-Src protein is overexpressed in 70% of BC tumors, and that in most of them c-Src is co-expressed with at least one ErbB family member [45]. The finding that the basal activation of HER2 and EGFR depends, in part, on the activity of other additional signaling pathways suggests that these instigator pathways might be used for therapeutic purposes to deregulate the activation of RTKs. We observed that overexpression of NK-1R in a BC cell line increases c-Src phosphorylation at Tyr416 more than 6-fold under the stimulus of SP, in addition to increasing HER2 phosphorylation. On the other hand, chemical inhibition of NK-1R decreases c-Src phosphorylation at Y416 in the BT-474 and SK-BR-3 cell lines and the combination of NK-1R, NK-2R and NK-3R chemical inhibitors strongly decreases c-Src phosphorylation at Y416 in SK-BR-3 (cell line expressing all 3 tachykinin receptors). Thus, the use of c-Src and MMP inhibitors allowed us to demonstrate that the SP-mediated transactivation of HER2 or EGFR depends, in part, on c-Src and MMP signaling pathways in BC cell lines. Moreover, the use of these inhibitors demonstrated that this Src and MMP-dependent signaling is important to the cell viability and migration capacity of HER2+ and EGFR+ cell lines, being more pronounced using NK1-R antagonist, L-733,060 alone or in combination. These results suggest an oncogenic addiction to NK-1R signaling in breast cancer cells, where c-Src and MMPs play an important role, probably due to the transactivation mechanism-dependent process of HER2 and EGFR.

Therefore, the c-Src protein may be crucial not only in the ligand-independent transactivation of RTKs, but probably also in MMP maintenance and activation by triggering cleavage of membrane-anchored ligands. These ligands, once released, would bind to receptors as EGFR [46, 47] which could homodimerize or heterodimerize with HER2 as the preferred heterodimerization partner.

In summary, we have shown that c-Src and MMPs are involved in HER2 and EGFR transactivation processes through NK-1R in BC. Therefore, a simultaneous blockade of ERBB receptors and other instigators of c-Src/MMP-induced MAPK activation such as NK-1R may improve treatment responses against the ERBB family of receptors.
